# Hesperidin depolarizes the pacemaker potentials through 5-HT_4_ receptor in murine small intestinal interstitial cells of Cajal

**DOI:** 10.1080/19768354.2020.1746398

**Published:** 2020-03-26

**Authors:** Minwoo Hwang, Jeong Nam Kim, Byung Joo Kim

**Affiliations:** a Department of Sasang Constitutional Medicine, College of Korean Medicine, Kyung Hee University, Seoul, Republic of Korea; b Division of Longevity and Biofunctional Medicine, Pusan National University School of Korean Medicine, Yangsan, Republic of Korea

**Keywords:** Hesperidin, interstitial cells of Cajal, gastrointestinal motility, pacemaker potential

## Abstract

Hesperidin, a citrus flavonoid, can exert numerous beneficial effects on human health. Interstitial cells of Cajal (ICC) are pacemaker cells in the gastrointestinal (GI) tract. In the present study, we investigated potential effects of hesperidin on pacemaker potential of ICC in murine small intestine and GI motility. A whole-cell patch-clamp configuration was used to record pacemaker potential in ICC, and GI motility was investigated *in vivo* by recording gastric emptying (GE) and intestinal transit rate (ITR). Hesperidin depolarized pacemaker potentials of ICC in a dose-dependent manner. Pre-treatment with methoctramine or 4-DAMP did not inhibit hesperidin-induced pacemaker potential depolarization. Neither a 5-HT_3_ receptor antagonist (Y25130) nor a 5-HT_7_ receptor antagonist (SB269970) reduced the effect of hesperidin on ICC pacemaker potential, whereas the 5-HT_4_ receptor antagonist RS39604 was found to inhibit this effect. In the presence of GDP–β–S, hesperidin-induced pacemaker potential depolarization was inhibited. Moreover, in the presence of U73122 and calphostin C, hesperidin did not depolarize pacemaker potentials. Furthermore, hesperidin accelerated GE and ITR *in vivo*. These results imply that hesperidin depolarized ICC pacemaker potential via 5-HT_4_ receptors, G protein, and PLC/PKC dependent pathways and that it increased GI motility. Therefore, hesperidin may be a promising novel drug to regulate GI motility.

## Introduction

Polyphenols occur in various plants and are important for their defense systems; flavonoids are a group of polyphenols which are particularly common in edible plants that constitute a large part of human diet (Cho et al. 2018; Chung et al. 2018; Stevens et al. 2019). The predominant flavonoid in sweet fruits is hesperidin (Garg et al. 2001) which also occurs frequently in vegetables and beverages such as tea and red wine (Orallo et al. 2004; Bock et al. 2008). Hesperidin is known to exert various effects on humans including anti-inflammatory activity (Garg et al. 2001). Moreover, hesperidin has been reported to regulate gastrointestinal (GI) motility by reducing inflammatory reactions and stimulating calcium release (Xiong et al. 2016).

GI motility is controlled by numerous different cell types in the GI tract, and among these, ICC plays a key role (Huizinga et al. 1995; Sanders 1996; Kim et al. 2005). ICC can generate electrical charges (Huizinga et al. 1995; Sanders 1996; Kim et al. 2005), and when ICC abundance and intercellular connectivity are reduced, GI motility also decreases (Der et al. 2000; Wei et al. 2014). Potential effects of hesperidin on GI motility have not yet been comprehensively investigated; therefore, in the current study, we assessed the effects of hesperidin on ICC *in vitro* and on GI motility *in vivo*.

## Materials and methods

### Preparation of cell cultures

Animal experiments were conducted in compliance with the stipulations of the animal experiment ethics committee of Pusan National University (approval no. PNU-2018-1832). Small intestines of ICR mice were isolated, and the mucous membrane was excised. Small-intestinal muscles were equilibrated using Ca^2+^-free Hank’s solution. Cells were enzymatically isolated using collagenase (Worthington Biochemical, Lakewood, NJ, USA) and were then cultured in smooth muscle growth medium ([SMGM]; Clonetics, San Diego, CA, USA) inside a CO_2_ incubator and at 37°C.

### Patch-clamp experiments

Na^+^-Tyrode solution was used in bath, and the solution was produced using KCl 140, MgCl_2_ 5, K_2_ATP 2.7, NaGTP 0.1, creatine phosphate disodium 2.5, HEPES 5, and EGTA 0.1. Electrophysiological analyses were conducted, and results were analyzed using pClamp (Molecular Devices, Sunnyvale, CA, USA) and Origin software (version 6.0, Microcal, USA).

### Assessment of gastric emptying (GE)

Twenty minutes after administering phenol red solution, stomachs tissue was cut into several pieces which were placed in sodium hydroxide. Tissue preparations were then centrifuged with NaOH at 1050 × *g* for 10 min, and absorbance was measured using a spectrometer at 560 nm, according to previously published methods.

### Intestinal transit rate (ITR) measurements

Mice were administered hesperidin, followed by oral administration of Evans Blue. Thirty minutes later, animals were euthanized, and ITR was measured according to the distance over which Evans Blue had been transported in the intestine (expressed as percentage of the length of the intestine).

### Drugs

5-HT receptor antagonists were obtained from Tocris Bioscience (Bristol, United Kingdom). All other reagents including hesperidin were purchased from Sigma-Aldrich (St. Louis, MO, USA).

### Statistical analyses

Results are shown as means ± standard error. We employed an ANOVA to test effects of hesperidin on the respective parameters using Prism 6.0 software (La Jolla, CA, USA). Statistical significance is reported at *P *< 0.05.

## Results

### Effect of hesperidin on pacemaker potentials in ICC

**Figure 1.**
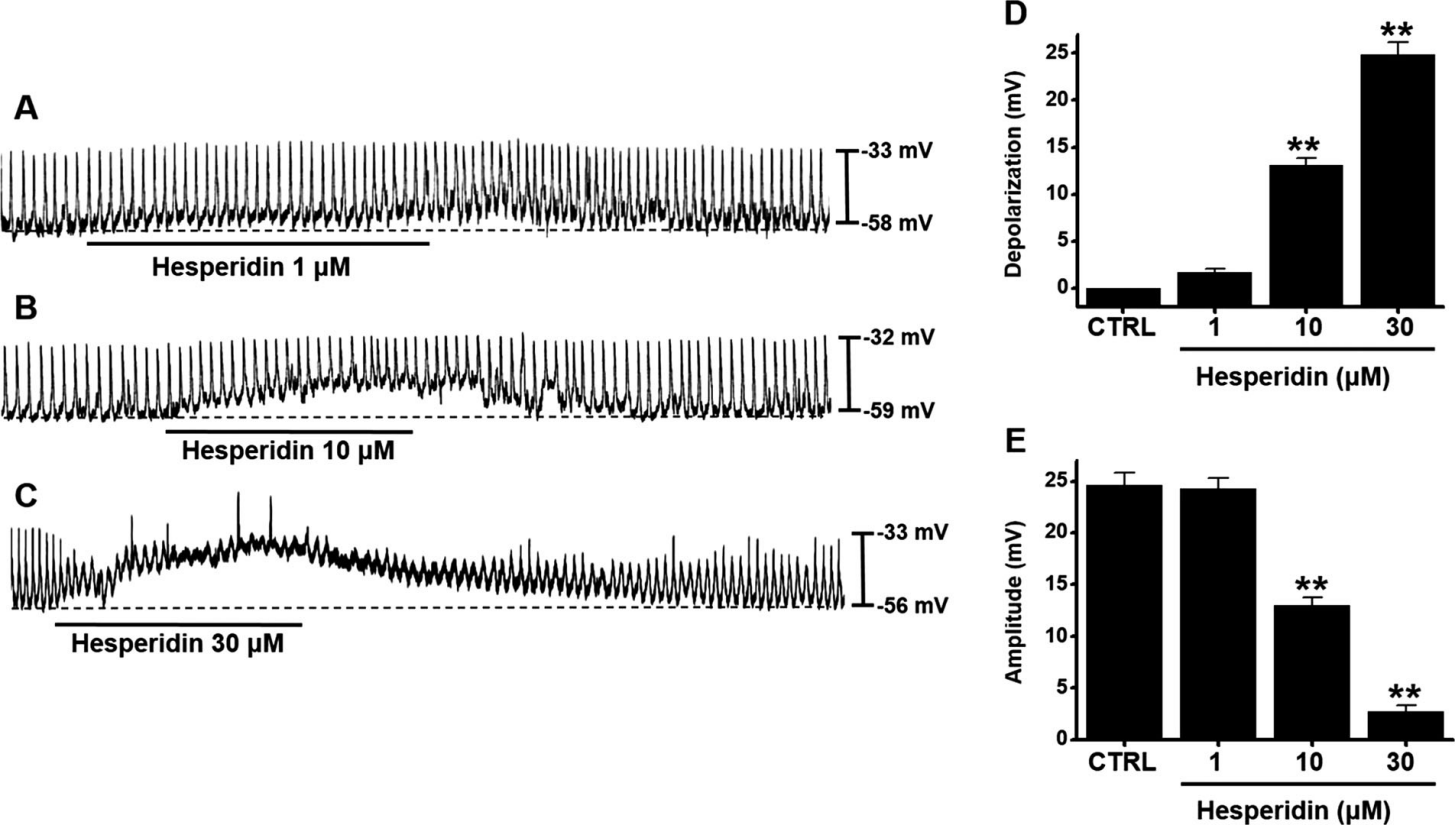
Effects of hesperidin on pacemaker potentials of murine small intestine ICC. (A–C) Hesperidin depolarized pacemaker potentials of ICC. (D and E) Summaries of pacemaker potential depolarization and amplitude changes due to hesperidin. Bars indicate mean values ± SEs. **P < 0.01. CTRL: Control.

Spontaneous pacemaker potentials were observed in ICC. The membrane potential was −56.3 ± 1.7 mV, and the amplitude was 26.6 ± 1.2 mV. Hesperidin depolarized pacemaker potentials in a dose-dependent manner (1–30 μM; Figure 1A–C). Values of depolarization were 1.7 ± 0.5 mV at 1 μM, 13.1 ± 0.7 mV (*P* < 0.01) at 10 μM, and 24.8 ± 1.3 mV (*P* < 0.01) at 30 μM (Figure 1D), and amplitude values were 24.3 ± 1.0 mV at 1 μM, 13.0 ± 0.7 mV (*P* < 0.01) at 10 μM, and 2.7 ± 0.6 mV (*P* < 0.01) at 30 μM (Figure 1E).

### Hesperidin-induced pacemaker potential depolarization in ICC and muscarinic receptors

**Figure 2.**
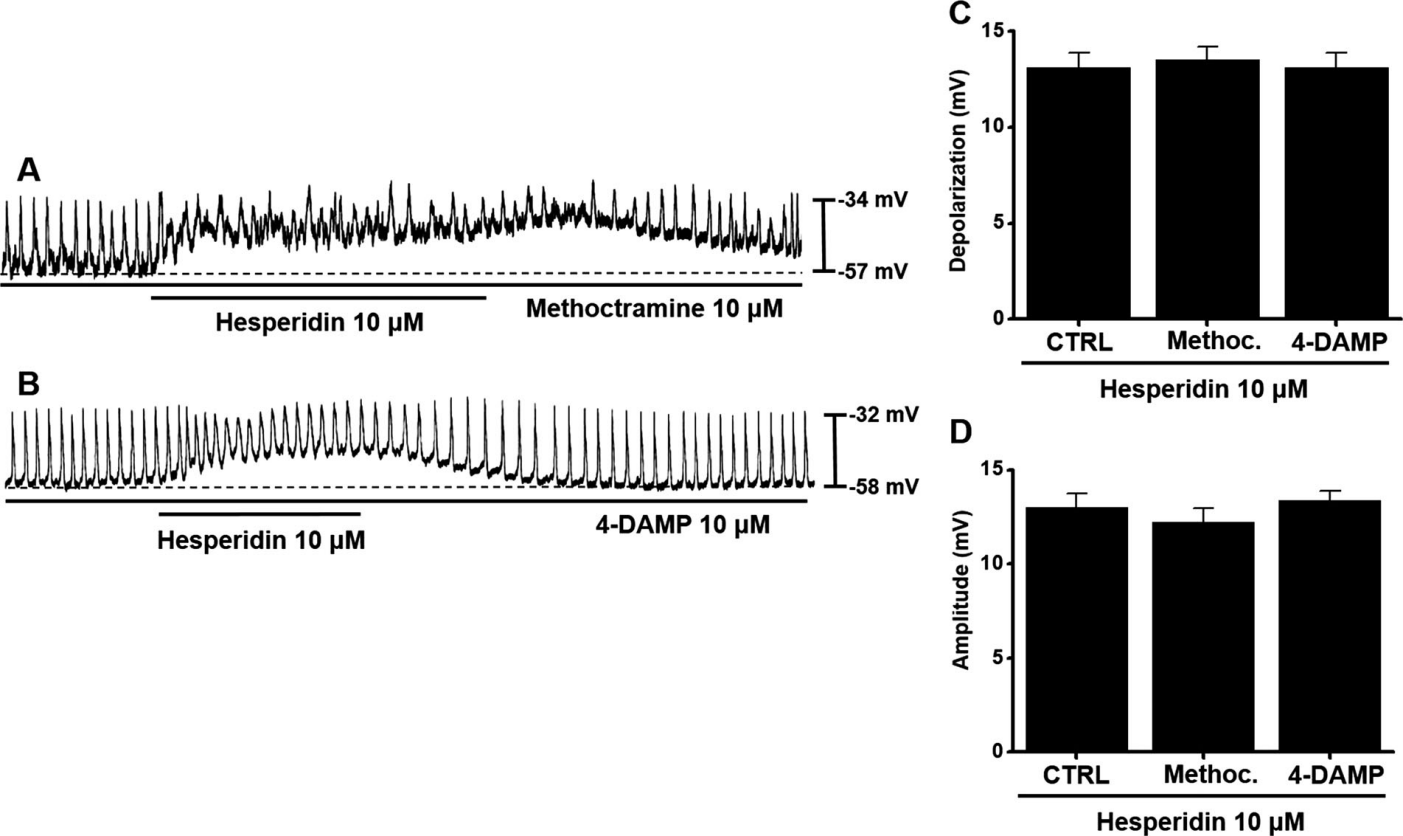
Effects of muscarinic receptor antagonists on hesperidin-induced pacemaker potential depolarization in ICC. (A) In presence of methoctramine, hesperidin depolarized pacemaker potentials of ICC. (B) With 4-DAMP, hesperidin depolarized pacemaker potentials of ICC. (C and D) Summaries of pacemaker potential depolarization and amplitude changes due to hesperidin with muscarinic receptor antagonists. Bars indicate mean values ± SEs. **P < 0.01. CTRL: Control. Methoc.: Methoctramine.

Previous studies suggested that muscarinic receptors affect excitatory nerve transmission in the GI tract (Huizinga et al. 1984; Inoue and Chen 1993). Moreover, M2 and M3 subtypes of muscarinic receptors occur in cultured ICC (Epperson et al. 2000). Therefore, we investigated involvement of M2 and M3 subtypes in hesperidin-induced pacemaker potential depolarization in ICC. Neither methoctramine (an M2 receptor antagonist) nor 4-DAMP (an M3 receptor antagonist) produced an effect on hesperidin-induced pacemaker potential depolarization (Figure 2A,B, respectively). Depolarization values were 13.5 ± 0.7 mV with methoctramine and 13.1 ± 0.8 mV with 4-DAMP (Figure 2C), and amplitude values were 12.2 ± 0.8 mV with methoctramine and 13.4 ± 0.5 mV with 4-DAMP (Figure 2D).

### Involvement of the 5-HT_4_ receptor in hesperidin-induced pacemaker potential depolarization in ICC

**Figure 3.**
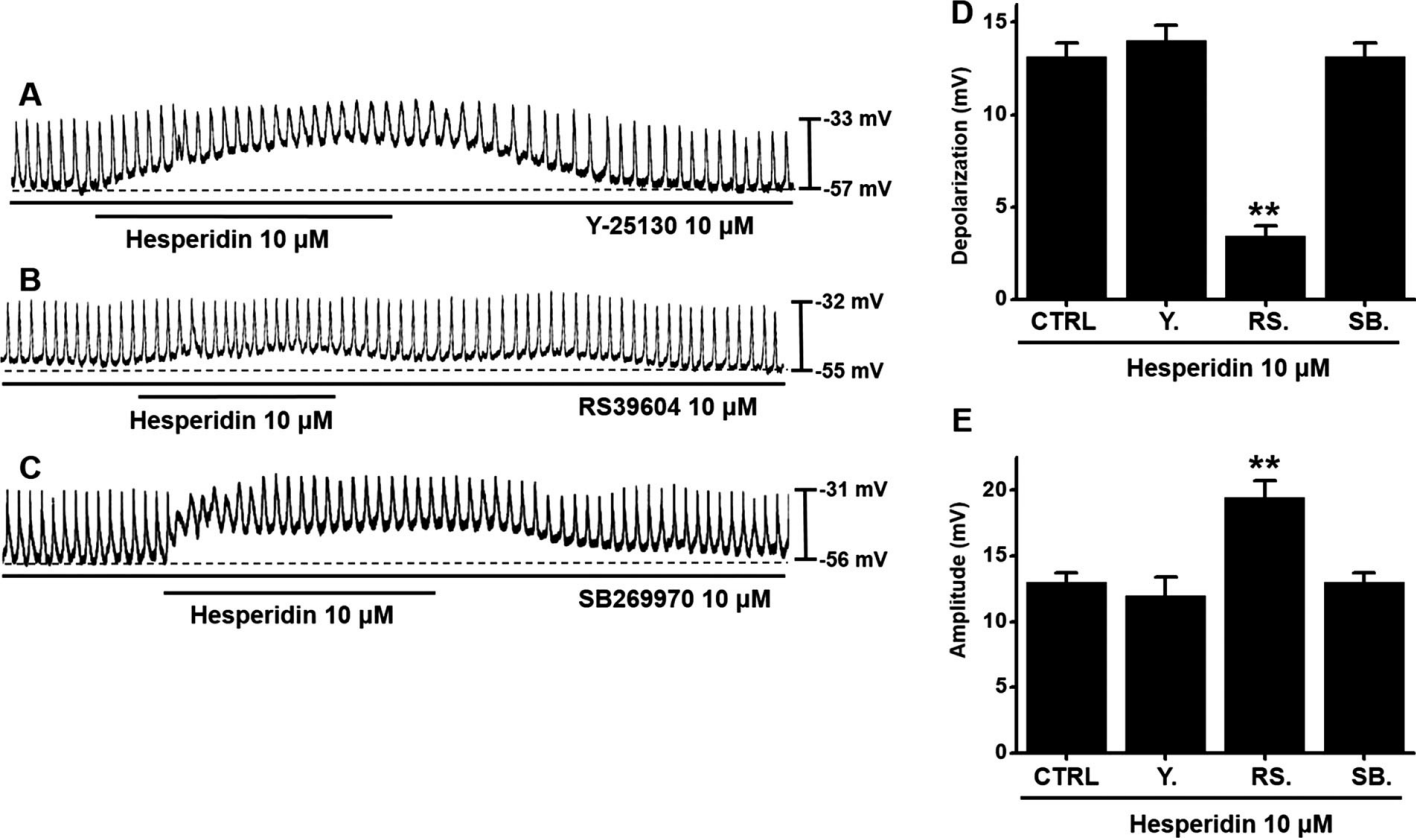
Effects of 5-HT receptor antagonists on hesperidin-induced pacemaker potential depolarization in ICC. (A) In presence of Y25130 (5-HT_3_ receptor antagonist), hesperidin depolarized pacemaker potentials of ICC. (B) In presence of RS39604 (a 5-HT_4_ receptor antagonist), hesperidin did not depolarize pacemaker potential of ICC. (C) In presence of SB269970 (a 5-HT_7_ receptor antagonist), hesperidin depolarized pacemaker potential of ICC. (D and E) Summaries of pacemaker potential depolarization and amplitude changes due to hesperidin with 5-HT receptor antagonists. Bars indicate mean values ± SEs. **P < 0.01. CTRL: Control. Y.: Y25130. RS.: RS39604. SB.: SB269970.

5-HT receptors are known to be involved in the function of GI motility, thus we investigated the involvement of 5-HT receptors (Gershon and Tack 2007). Previous studies found that only 5-HT_3,4,7_ R were expressed (Liu et al. 2011; Shahi et al. 2011). Neither 5-HT_3_ receptor antagonist Y25130 nor 5-HT_7_ receptor antagonist SB269970 showed any effect on hesperidin-induced responses (Figure 3A,C); however, 5-HT_4_ receptor antagonist RS39604 inhibited the effect of hesperidin on pacemaker potentials of ICC (Figure 3B). In the presence of hesperidin and 5-HT receptor antagonists, depolarization values were 14.0 ± 0.8 mV with Y25130, 13.4 ± 0.6 mV (*P* < 0.01) with RS39604, and 13.1 ± 0.8 mV with SB269970 (Figure 3D), and mean amplitude values were 12.1 ± 1.4 mV with Y25130, 19.5 ± 1.3 mV (*P* < 0.01) with RS39604, and 13.0 ± 0.7 mV with SB269970 (Figure 3E).

### Involvement of G proteins in hesperidin-induced pacemaker potential depolarization in ICC

**Figure 4.**
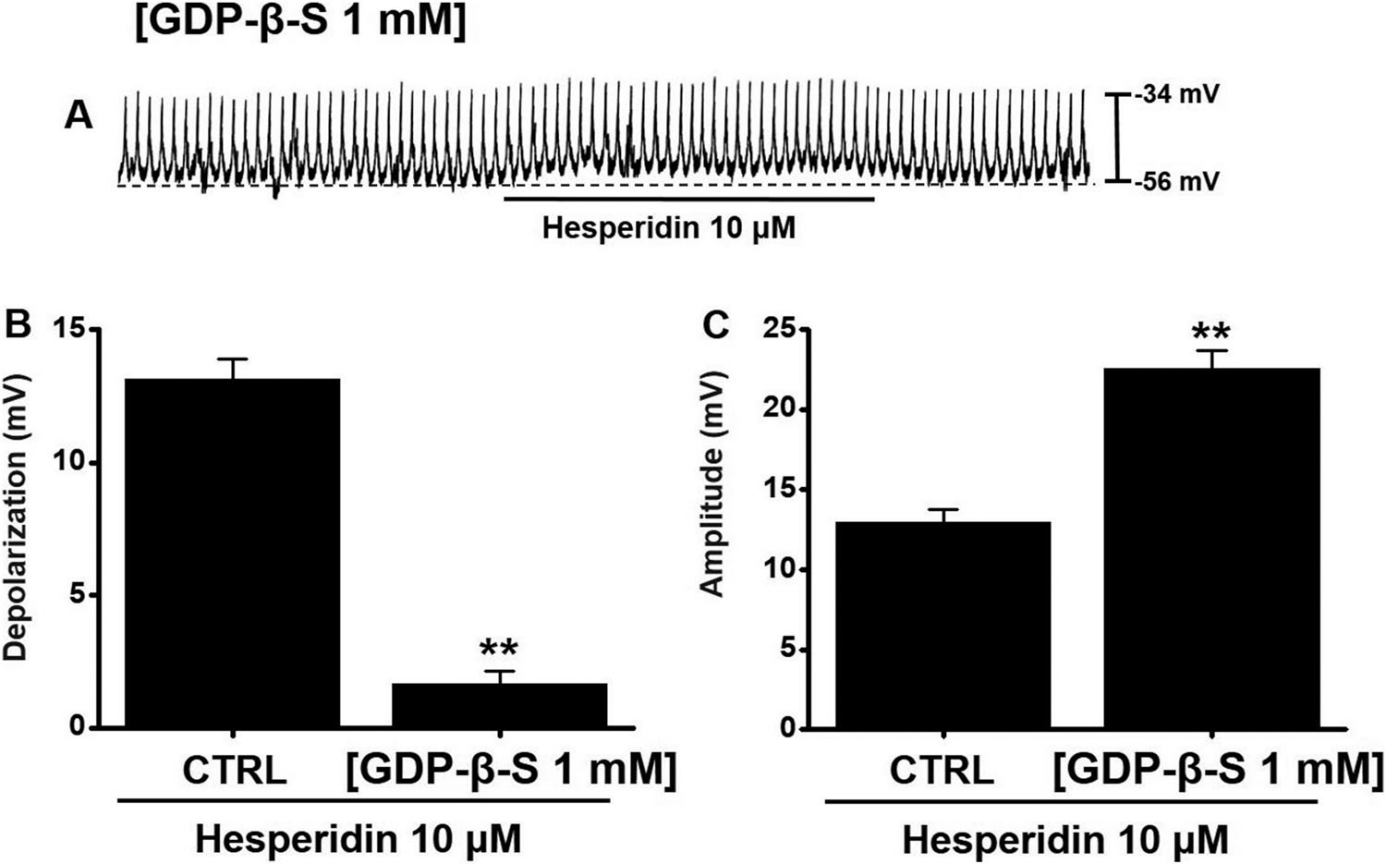
Effects of GDP–β–S on hesperidin–induced pacemaker potential depolarization in ICC. (A) In presence of GDP–β–S (1 mM), hesperidin had no effects. (B and C) Summaries of pacemaker potential depolarization and amplitude changes due to hesperidin with GDP–β–S. Bars indicate mean values ± SEs. **P < 0.01. CTRL: Control.

GDP–β–S were used to inactivate G–protein (Komori et al. 1992; Ogata et al. 1996). When GDP–β–S occurred in the cell, hesperidin-induced pacemaker potential depolarization was inhibited (Figure 4A). In presence of GDP–β–S, a depolarization value of 1.7 ± 0.5 mV was observed (*P* < 0.01; Figure 4B), and the mean amplitude value was 22.6 ± 1.1 mV (*P* < 0.01) (Figure 4C).

### Involvement of phospholipase C (PLC), protein kinase A (PKA), and protein kinase C (PKC) in hesperidin-induced pacemaker potential depolarization in ICC

**Figure 5.**
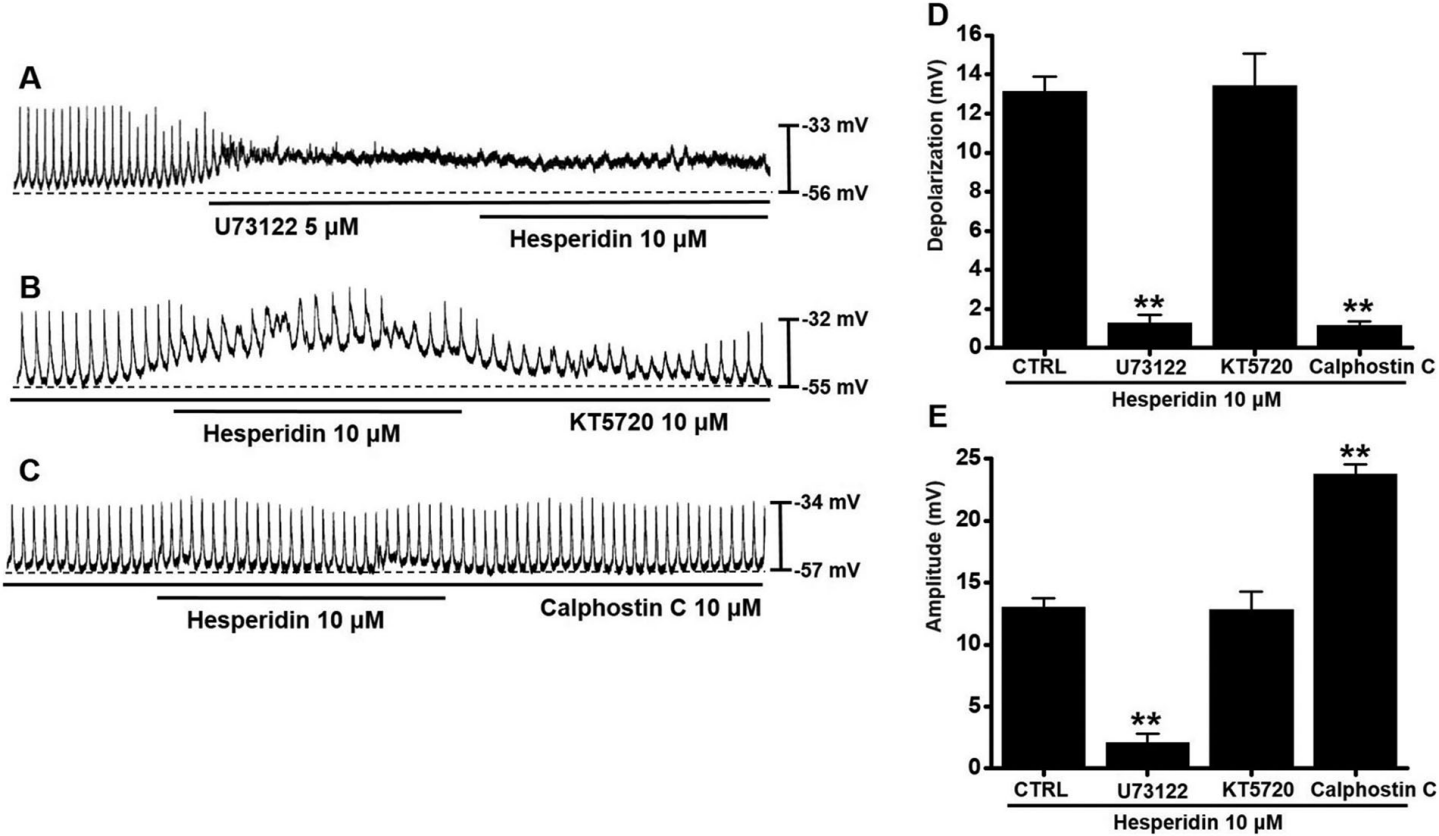
Effects of phospholipase C (PLC), protein kinase A (PKA), and protein kinase C (PKC) inhibitors on hesperidin-induced pacemaker potential depolarization in ICC. (A) In the presence of U73122 (a PLC inhibitor), hesperidin showed no effect. (B) In the presence of KT5720 (a PKA inhibitor), hesperidin depolarized pacemaker potential of ICC. (C) In the presence of calphostin C (a PKC inhibitor), hesperidin showed no effect. (D and E) Summaries of pacemaker potential depolarization and amplitude changes due to hesperidin with PLC, PKA, or PKC inhibitors. Bars indicate mean values ± SEs. **P < 0.01. CTRL: Control.

To investigate the involvement of PLC, PKA, and PKC pathways, we used U73122 (a PLC inhibitor), KT5720 (a PKA inhibitor), and calphostin C (a PKC inhibitor). Neither U73122 nor calphostin C produced an effect on hesperidin-associated responses (Figure 5A,C); however, in the presence of KT5720, hesperidin was depolarized (Figure 5B). In the presence of U73122, KT5720, or calphostin C, depolarization values were 1.3 ± 0.4 mV (*P* < 0.01) with U73122, 13.4 ± 1.7 mV with KT5720, and 1.1 ± 0.2 mV (*P* < 0.01) with calphostin C (Figure 5D), and amplitude values were 2.1 ± 0.7 mV (*P* < 0.01) with U73122, 12.8 ± 1.5 mV with KT5720, and 23.7 ± 0.8 mV (*P* < 0.01) with calphostin C (Figure 5E).

### Effects of hesperidin on GE

**Figure 6.**
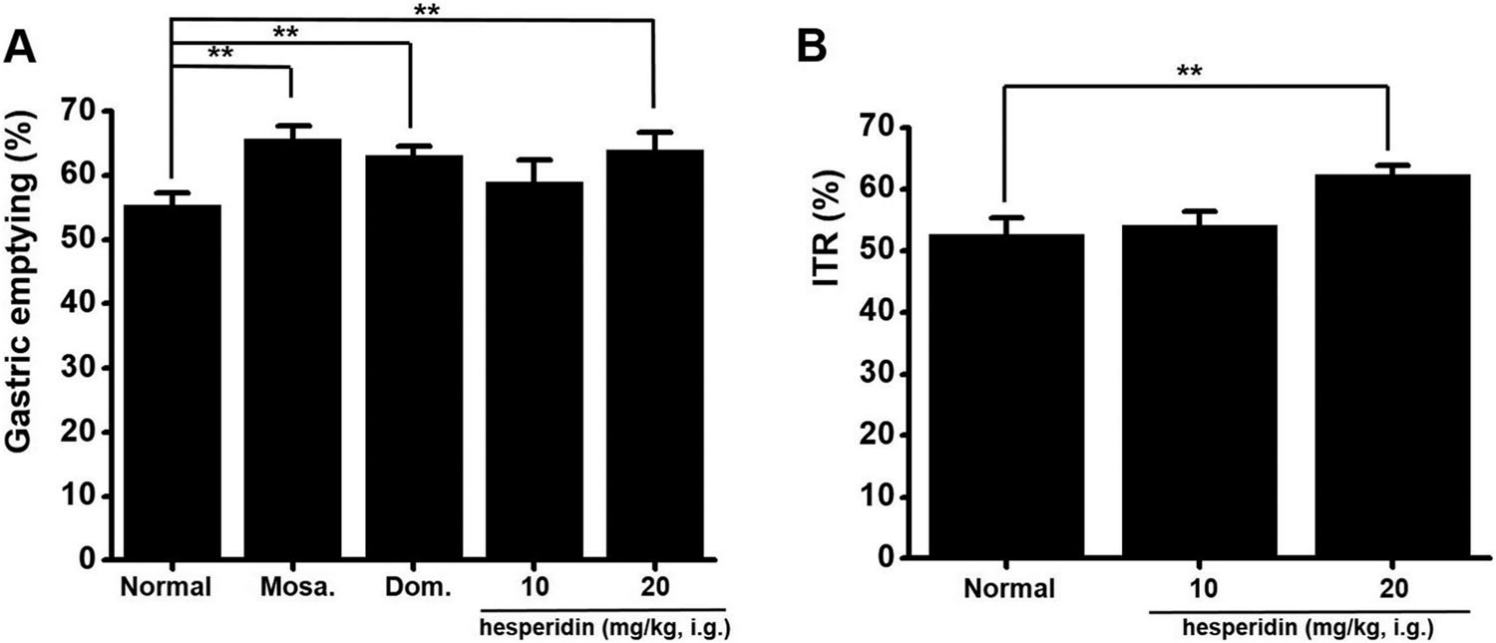
Effect of hesperidin on gastric emptying (GE) and intestinal transit rates (ITR). (A) For comparison, we used mosapride and domperidone. Hesperidin increased GE values. (B) Hesperidin accelerated ITR. Bars indicate mean values ± SEs. **P < 0.01. CTRL: Control. Mosa.: Mosapride; Dom.: Domperidone. i.g.: intragastric.

Effects of hesperidin were compared with those of mosapride and domperidone. Mice treated with hesperidin (10 and 20 mg/kg) showed higher GE values than controls (55.4 ± 1.9%). GE values in hesperidin treatments were 58.9 ± 3.4% at 10 mg/kg and 63.9 ± 2.8% at 20 mg/kg (*P *< 0.01; Figure 6A). The GE values of mosapride and domperidone were 65.7 ± 2.0% (*P *< 0.01) and 63.1 ± 1.4% (*P *< 0.01; Figure 6A), respectively.

### Effects of hesperidin on ITR

The mean ITR value in untreated mice, 10 mg/kg hesperidin treatment, and 20 mg/kg hesperidin treatment was 52.7 ± 2.7%, 54.1 ± 2.3%, and 62.3 ± 1.6%, respectively (*P *< 0.01; Figure 6B).

## Discussion

We investigated effects of hesperidin on pacemaker potential in ICC and on GI motility. Hesperidin depolarized pacemaker potentials through 5-HT_4_ receptors via G protein and PLC/PKC dependent pathways but not through muscarinic receptors, indicating that hesperidin can modulate ICC. Furthermore, hesperidin increased GE and ITR.

Hesperidin is commonly found in highly nutritious fruits such as oranges, tangelos, tangerines, grapefruits, and other citrus fruits (Suzuki et al. 2014). Hesperidin has been shown to exert numerous biological functions (Hou et al. 2012); therefore, hesperidin has been commonly used to treat various disorders including neurological, psychiatric, and cardiovascular diseases (Li and Schluesener 2017). Furthermore, hesperidin is being used in combination with other drugs such as rikkunshito (Li and Schluesener 2017). Rikkunshito is a traditional herbal remedy to promote appetite and is commonly used in Japan to treat indigestion (Arai et al. 2012; Takiguchi et al. 2013). Hesperidin stimulates ghrelin secretion, thus synergistic effects of mixing hesperidin with rikkunshito produced promising good results. In addition, hesperidin is readily available and inexpensive, therefore it can be manufactured and commercialized for therapeutic purposes as well as in the food industry. In the present study, we found that hesperidin modulated pacemaker potentials in ICC. Therefore, we suggest that hesperidin may regulate GI motility via ICC pacemaker potential.

GI motility disorders are very common and occur throughout the GI tract, and the associated symptoms can substantially affect the quality of life (Pare et al. 2001; El-Serag and Talley 2003; Lacy and Weiser 2006; Lacy et al. 2018). Moreover, these disorders are of substantial economical concern (Sandler et al. 2002; Lacy et al. 2018). ICC are important for GI motility (Huizinga et al. 1995; Sanders 1996; Kim et al. 2005). ICC spontaneously generate active pacemaker potential, causing electrical and mechanical activity of smooth muscles (Huizinga et al. 1995; Sanders 1996; Kim et al. 2005). In ICC, a Ca^2+^-activated Cl^−^ channel and a non-selective cation channel are required for pacemaking activity, and the ether-a-go-go-related K^+^ channel is also one of the most important K^+^ channels for maintaining and activating stable membrane voltage in ICC (Zhu et al. 2003; Kim et al. 2005; Zhu et al. 2009). Further studies on the involvement of ion channels in hesperidin-related effects are required.

Muscarinic receptors are important receptors for regulating GI motility (Hirst et al. 2002). In the present study, both methoctramine and 4-DAMP produced no effects on hesperidin reactions. Thus, muscarinic receptors were apparently not involved in hesperidin effects on ICC (Figure 2). Furthermore, 5-HT plays a crucial role in regulating GI motility (Baker 2005). Previous studies suggested that 5-HT can control pacemaker activity through 5-HT_3_, 5-HT_4_, and 5-HT_7_ receptors (Shahi et al. 2011). In the present study, RS39604 blocked hesperidin effects, whereas Y25130 and SB269970 showed no respective effect. Thus, 5-HT_4_ receptors were apparently involved in hesperidin-induced effects on ICC (Figure 3). Furthermore, G-protein and PLC/PKC pathways are involved in hesperidin-induced effects in ICC (Figures 4 and 5). In addition, we found that hesperidin increased GE and ITR (Figure 6).

Polyphenols occur naturally in various fruits and vegetables. However, potential effects of polyphenols on human health remain to be elucidated. Our results suggest that polyphenols such as hesperidin may be an option for prevention and treatment of GI disorders. Taken together, our results show that hesperidin depolarizes pacemaker potentials of ICC via 5-HT_4_ receptors, G protein, and PLC/PKC dependent pathways, and increases GE and ITR.

## References

[CIT0001] Arai M, Matsumura T, Tsuchiya N, Sadakane C, Inami R, Suzuki T, Yoshikawa M, Imazeki F, Yokosuka O. 2012. Rikkunshito improves the symptoms in patients with functional dyspepsia, accompanied by an increase in the level of plasma ghrelin. Hepato-Gastroenterology. 59:62–66. 22260823 10.5754/hge11246

[CIT0002] Baker DE. 2005. Rationale for using serotonergic agents to treat irritable bowel syndrome. Am J Health Syst Pharm. 62:700–711. doi: 10.1093/ajhp/62.7.700 15790796

[CIT0003] Bock C, Waldmann KH, Ternes W. 2008. Mangiferin and hesperidin metabolites are absorbed from the gastrointestinal tract of pigs after oral ingestion of a Cyclopia genistoides (honeybush tea) extract. Nutr Res. 28:879–891. doi: 10.1016/j.nutres.2008.08.001 19083501

[CIT0004] Cho J, Kweon H, Huh S, Sadra A. 2018. Augmented reduction in colonic inflammatory markers of dextran sulfate sodium induced colitis with a combination of 5-aminosalicylic acid and AD-lico™ from Glycyrrhiza inflate. Anim Cells Syst. 22:189–196. doi: 10.1080/19768354.2018.1476409 PMC6138317 30460097

[CIT0005] Chung HJ, Noh Y, Kim MS, Jang A, Lee CE, Myung SC. 2018. Steroidogenic effects of Taraxacum officinale extract on the levels of steroidogenic enzymes in mouse Leydig cells. Anim Cells Syst. 22:407–414. doi: 10.1080/19768354.2018.1494628 PMC6282421 30533263

[CIT0006] Der T, Bercik P, Donnelly G, Jackson T, Berezin I, Collins SM, Huizinga JD. 2000. Interstitial cells of Cajal and inflammation-induced motor dysfunction in the mouse small intestine. Gastroenterology. 119:1590–1599. doi: 10.1053/gast.2000.20221 11113080

[CIT0007] El-Serag HB, Talley NJ. 2003. Health-related quality of life in functional dyspepsia. Aliment Pharmacol Ther. 18:387–393. doi: 10.1046/j.1365-2036.2003.01706.x 12940923

[CIT0008] Epperson A, Hatton WJ, Callaghan B, Doherty P, Walker RL, Sanders KM, Ward SM, Horowitz B. 2000. Molecular markers expressed in cultured and freshly isolated interstitial cells of Cajal. Am J Physiol Cell Physiol. 279:C529–C539. doi: 10.1152/ajpcell.2000.279.2.C529 10913020

[CIT0009] Garg A, Garg S, Zaneveld LJ, Singla AK. 2001. Chemistry and pharmacology of the citrus bioflavonoid hesperidin. Phytother Res. 15:655–669. doi: 10.1002/ptr.1074 11746857

[CIT0010] Gershon MD, Tack J. 2007. The serotonin signaling system: from basic understanding to drug development for functional GI disorders. Gastroenterology. 132:397–414. doi: 10.1053/j.gastro.2006.11.002 17241888

[CIT0011] Hirst GD, Dickens EJ, Edwards FR. 2002. Pacemaker shift in the gastric antrum of guinea-pigs produced by excitatory vagal stimulation involves intramuscular interstitial cells. J Physiol. 541:917–928. doi: 10.1113/jphysiol.2002.018614 12068050 PMC2290357

[CIT0012] Hou M, Man M, Man W, Zhu W, Hupe M, Park K, Crumrine D, Elias PM, Man MQ. 2012. Topical hesperidin improves epidermal permeability barrier function and epidermal differentiation in normal murine skin. Exp Dermatol. 21:337–340. doi: 10.1111/j.1600-0625.2012.01455.x 22509829 PMC3335754

[CIT0013] Huizinga JD, Chang G, Diamant NE, El-Sharkawy TY. 1984. Electrophysiological basis of excitation of canine colonic circular muscle by cholinergic agents and substance P. J Pharmacol Exp Ther. 231:692–699. 6209389

[CIT0014] Huizinga JD, Thuneberg L, Kluppel Malysz MJ, Mikkelsen HB, Bernstein A. 1995. W/kit gene required for interstitial cells of Cajal and for intestinal pacemaker activity. Nature. 373:347–349. doi: 10.1038/373347a0 7530333

[CIT0015] Inoue R, Chen S. 1993. Physiology of muscarinic receptor operated nonselective cation channels in guinea-pig ileal smooth muscle. Exs. 66:261–268. 7505656 10.1007/978-3-0348-7327-7_20

[CIT0016] Kim BJ, Lim HH, Yang DK, Jun JY, Chang IY, Park CS, So I, Stanfield PR, Kim KW. 2005. Melastatin-type transient receptor potential channel 7 is required for intestinal pacemaking activity. Gastroenterology. 129:1504–1517. doi: 10.1053/j.gastro.2005.08.016 16285951

[CIT0017] Komori S, Kawai M, Takewaki T, Ohashi H. 1992. GTP-binding protein involvement in membrane currents evoked by carbachol and histamine in Guinea-pig ileal muscle. J Physiol. 450:105–126. doi: 10.1113/jphysiol.1992.sp019118 1432705 PMC1176113

[CIT0018] Lacy BE, Crowell MD, Mathis C, Bauer D, Heinberg LJ. 2018. Gastroparesis: quality of life and health care utilization. J Clin Gastroenterol. 52:20–24. doi: 10.1097/MCG.0000000000000728 27775961

[CIT0019] Lacy BE, Weiser K. 2006. Gastrointestinal motility disorders: an update. Dig Dis. 24:228–242. doi: 10.1159/000092876 16849850

[CIT0020] Li C, Schluesener H. 2017. Health-promoting effects of the citrus flavanone hesperidin. Crit Rev Food Sci Nutr. 57:613–631. doi: 10.1080/10408398.2014.906382 25675136

[CIT0021] Liu HN, Ohya S, Nishizawa Y, Sawamura YK, Iino S, Syed MM, Goto K, Imaizumi Y, Nakayama S. 2011. Serotonin augments gut pacemaker activity via 5-HT3 receptors. PLoS One. 6:e24928. doi: 10.1371/journal.pone.0024928 21949791 PMC3174222

[CIT0022] Ogata R, Inoue Y, Nakano H, Ito Y, Kitamura K. 1996. Oestradiol-induced relaxation of rabbit basilar artery by inhibition of voltage-dependent Ca channels through GTP-binding protein. Br J Pharmacol. 117:351–359. doi: 10.1111/j.1476-5381.1996.tb15198.x 8789390 PMC1909250

[CIT0023] Orallo F, Alvarez E, Basaran H, Lugnier C. 2004. Comparative study of the vasorelaxant activity, superoxide-scavenging ability and cyclicnucleotide phosphodiesterase-inhibitory effects of hesperetin and hesperidin. Naunyn Schmiedebergs Arch Pharmacol. 370:452–463. doi: 10.1007/s00210-004-0994-6 15599707

[CIT0024] Pare P, Ferrazzi S, Thompson WG, Irvine EJ, Rance L. 2001. An epidemiological survey of constipation in Canada: definitions, rates, demographics, and predictors of health care seeking. Am J Gastroenterol. 96:3130–3137. doi: 10.1111/j.1572-0241.2001.05259.x 11721760

[CIT0025] Sanders KM. 1996. A case for interstitial cells of Cajal as pacemakers and mediators of neurotransmission in the gastrointestinal tract. Gastroenterology. 111:492–515. doi: 10.1053/gast.1996.v111.pm8690216 8690216

[CIT0026] Sandler RS, Everhart JE, Donowitz M, Adams E, Cronin K, Goodman C, Gemmen E, Shah S, Avdic A, Rubin R. 2002. The burden of selected digestives diseases in the United States. Gastroenterology. 122:1500–1511. doi: 10.1053/gast.2002.32978 11984534

[CIT0027] Shahi PK, Choi S, Zuo DC, Yeum CH, Yoon PJ, Lee J, Kim YD, Park CG, Kim MY, Shin HR, et al. 2011. 5-hydroxytryptamine generates tonic inward currents on pacemaker activity of interstitial cells of cajal from mouse small intestine. Korean J Physiol Pharmacol. 15:129–135. doi: 10.4196/kjpp.2011.15.3.129 21860590 PMC3154376

[CIT0028] Stevens Y, Rymenant EV, Grootaert C, Camp JV, Possemiers S, Masclee A, Jonkers D. 2019. The intestinal fate of citrus flavanones and their effects on gastrointestinal health. Nutrients. 11:1464. doi: 10.3390/nu11071464 PMC6683056 31252646

[CIT0029] Suzuki H, Asakawa A, Kawamura N, Yagi T, Inui A. 2014. Hesperidin potentiates ghrelin signaling. Recent Pat Food Nutr Agric. 6:60–63. doi: 10.2174/2212798406666140825120623 25176345

[CIT0030] Takiguchi S, Hiura Y, Takahashi T, Kurokawa Y, Yamasaki M, Nakajima K, Miyata H, Mori M, Hosoda H, Kangawa K, et al. 2013. Effect of rikkunshito, a Japanese herbal medicine, on gastrointestinal symptoms and ghrelin levels in gastric cancer patients after gastrectomy. Gastric Cancer. 16:167–174. doi: 10.1007/s10120-012-0164-3 22895614

[CIT0031] Wei J, Li N, Xia X, Chen X, Peng F, Besner GE, Feng J. 2014. Effects of lipopolysaccharide induced inflammation on the interstitial cells of Cajal. Cell Tissue Res. 356:29–37. doi: 10.1007/s00441-013-1775-7 24435644

[CIT0032] Xiong YJ, Chu HW, Lin Y, Han F, Li YC, Wang AG, Wang FJ, Chen DP, Wang JY. 2016. Hesperidin alleviates rat postoperative ileus through anti-inflammation and stimulation of Ca(2+)-dependent myosin phosphorylation. Acta Pharmacol Sin. 37:1091–1100. doi: 10.1038/aps.2016.56 27345626 PMC4973386

[CIT0033] Zhu Y, Golden CM, Ye J, Wang XY, Akbarali HI, Huizinga JD. 2003. ERG k+ currents regulate pacemaker activity in ICC. Am J Physiol Gastrointest Liver Physiol. 285:G1249–G1258. doi: 10.1152/ajpgi.00149.2003 12958021

[CIT0034] Zhu MH, Kim TW, Ro S, Yan W, Ward SM, Koh SD, Sanders KM. 2009. A Ca(2+)-activated Cl(−) conductance in interstitial cells of Cajal linked to slow wave currents and pacemaker activity. J Physiol. 587:4905–4918. doi: 10.1113/jphysiol.2009.176206 19703958 PMC2770155

